# Common peroneal nerve palsy after TKA in valgus deformities; a systematic review

**DOI:** 10.1186/s40634-021-00443-x

**Published:** 2022-01-20

**Authors:** Raymond Puijk, Rachid Rassir, Laura M. Kok, Inger N. Sierevelt, Peter A. Nolte

**Affiliations:** 1 Department of Orthopaedic Surgery, Spaarne Gasthuis, Spaarnepoort 1, 2130AT, Hoofddorp, the Netherlands; 2 Specialized Center of Orthopedic Research and Education (SCORE), Xpert Orthopedie, Amsterdam, the Netherlands

**Keywords:** Valgus deformity, Total knee arthroplasty, Soft tissue release, Peroneal nerve release, Common peroneal nerve palsy

## Abstract

**Purpose:**

The aim of this systematic review is to investigate the prevalence of Common Peroneal Nerve Palsy after total knee arthroplasty in valgus deformities. Furthermore, the effectiveness of a peroneal nerve release prior to arthroplasty to prevent the palsy will be investigated.

**Methods:**

PubMed and Google Scholar were searched. Search terms regarding valgus deformity and total knee arthroplasty were used. Data analysis and extraction were performed using the web application ‘Rayyan QCRI’ according to PRISMA guidelines and screened according to the inclusion and exclusion criteria.

**Results:**

Twenty-seven studies were included, representing 1397 valgus knees. Knee balancing was performed in 19 studies with lateral soft tissue releases (1164 knees) and 8 studies (233 knees) with an additional osteotomy. Two studies (41 knees) in the lateral soft tissue release group conducted a peroneal nerve release simultaneous to arthroplasty. Common peroneal nerve palsies occurred in 26 cases (1.9%). Overall, no significant difference in palsy ratio between studies was found by using a peroneal nerve release (*p* = 0.90), between lateral soft tissue releases and osteotomies (*p* = 0.11) or between releases of specific ligaments.

**Conclusion:**

Common peroneal nerve palsies occur in 1.9% of the cases after total knee arthroplasty in valgus deformities. No difference in the number of palsies was seen when using a peroneal nerve release or using different balancing techniques. However, literature about peroneal nerve releases was very limited, therefore, the effectiveness of a peroneal nerve release remains unclear.

**Level of evidence:**

LEVEL III: Systematic review.

**Supplementary Information:**

The online version contains supplementary material available at 10.1186/s40634-021-00443-x.

## Introduction

Common peroneal nerve palsy (CPNP) is a feared complication after total knee arthroplasty (TKA). Previous studies show valgus deformity and flexion contracture as predisposing factors to develop CPNP [1–5]. An increased anatomical femorotibial angle (aFTA) of > 10° is commonly used to define a valgus knee [6–8]. In the literature, the reported CPNP incidence after TKA in valgus deformities with an aFTA > 10° (TKA-V) ranges between 0.3–9.5% [3, 9–17]. Injury of the common peroneal nerve (CPN) can be caused by indirect damage due to stretch or ischaemia after correction, or by direct injury due to laceration of the CPN during lateral soft tissue release (STR) [8, 11, 12, 18–20]. Commonly contracted and released ligaments in valgus knees include the iliotibial band (ITB), posterolateral capsule (PLC), lateral collateral ligament (LCL), popliteus tendon (POP) and lateral gastrocnemius tendon [8, 21]. The selection of ligaments to be released depend mainly on the tightness of the ligaments in extension and/or flexion. Ligaments can be released through pie-crusting [7, 13, 20, 22–25], a subperiosteal release [13, 23, 26–28] or in a transverse manner. Also, shifting the insertion of a ligament by use of an osteotomy (OT), like a lateral femoral epicondyle osteotomy (LFEO) [29–37] or medial femoral epicondyle osteotomy (MFEO) [30, 33] is used. However, an overzealous release may result in direct injury of the CPN, late-onset instability and even a higher revision rate [33, 38, 39].

Recovery from CPNP usually take place within a year; however, residual damage is certainly not uncommon [1, 4, 5, 12]. As CPNP has serious consequences, orthopaedic surgeons aim to prevent this complication by a concomitant peroneal nerve release (PNR) [8, 40]. A PNR is a procedure performed simultaneously to TKA-V, which explores the nerve and removes the constricted dressings to release the CPN. Therefore, it yields the nerve to have more capacity to extend and protects it against mechanical stretching after balancing the knee properly during TKA-V. Due to the limited number of studies investigating PNR, no consensus has yet been reached on the value and indication of the procedure.

This systematic review primarily attempts to investigate the CPNP incidence after TKA-V and the rate will be compared between different valgus correcting techniques, including lateral STR and OT. Secondarily, the effectiveness of a PNR in preventing CPNP after TKA-V will be investigated.

## Material and methods

### Search strategy

A librarian-assisted comprehensive search of the literature was performed in October 2020 in PubMed and Google Scholar. The primary search was mainly focused on the surgical treatment and outcome of TKA-V. Search terms and associated synonyms that were included in the search are displayed in Table 1. A total of 3.945 articles were identified through PubMed and Google Scholar (Fig. 1 [Additional file 1]). The analysis was done according to the ‘Preferred Reporting Items of Systematic review and Meta-analyses’ (PRISMA) [41]. Through the web application ‘Rayyan QCRI’ [42], duplicates were removed and the remaining articles were screened for eligibility, according to the screening criteria (Table 2). The screening was independently done by three reviewers (X, X and X), a fourth reviewer (X) was consulted in case of doubt about the suitability of an article [43]. To ensure no relevant articles were omitted, a cross-reference check was performed on the included articles. A consensus was achieved on all included articles based on inclusion and exclusion criteria.

**Table 1:** PubMed search strategy, October 2020

(((((((((“arthroplasty, replacement, knee”[MeSH Terms] OR ((“arthroplasty”[All Fields] AND “replacement”[All Fields]) AND “knee”[All Fields])) OR “knee replacement arthroplasty”[All Fields]) OR ((“total”[All Fields] AND “knee”[All Fields]) AND “arthroplasty”[All Fields])) OR “total knee arthroplasty”[All Fields]) OR ((((“arthroplasty, replacement, knee”[MeSH Terms] OR ((“arthroplasty”[All Fields] AND “replacement”[All Fields]) AND “knee”[All Fields])) OR “knee replacement arthroplasty”[All Fields]) OR ((“total”[All Fields] AND “knee”[All Fields]) AND “replacement”[All Fields])) OR “total knee replacement”[All Fields])) OR ((((“arthroplasty, replacement, knee”[MeSH Terms] OR ((“arthroplasty”[All Fields] AND “replacement”[All Fields]) AND “knee”[All Fields])) OR “knee replacement arthroplasty”[All Fields]) OR (“knee”[All Fields] AND “arthroplasty”[All Fields])) OR “knee arthroplasty”[All Fields])) OR ((((“arthroplasty, replacement, knee”[MeSH Terms] OR ((“arthroplasty”[All Fields] AND “replacement”[All Fields]) AND “knee”[All Fields])) “knee replacement arthroplasty”[All Fields]) OR (“knee”[All Fields] AND “replacement”[All Fields])) OR “knee replacement”[All Fields])) OR (“TKA”[Title/Abstract] OR “TKR”[Title/Abstract])) ((((((“genu valgum”[MeSH Terms] OR (“genu”[All Fields] AND “valgum”[All Fields])) OR “genu valgum”[All Fields]) OR (“genu”[All Fields] AND “valga”[All Fields])) OR “genu valga”[All Fields]) OR ((((“genu valgum”[MeSH Terms] OR (“genu”[All Fields] AND “valgum”[All Fields])) OR “genu valgum”[All Fields]) OR (“knock”[All Fields] AND “knee”[All Fields])) OR “knock knee”[All Fields])) OR “knock knees”[Title/Abstract])) (((((((((“arthroplasty, replacement, knee”[MeSH Terms] OR ((“arthroplasty”[All Fields] AND “replacement”[All Fields]) AND “knee”[All Fields])) OR “knee replacement arthroplasty”[All Fields]) OR ((“total”[All Fields] AND “knee”[All Fields]) AND “arthroplasty”[All Fields])) OR “total knee arthroplasty”[All Fields]) OR ((((“arthroplasty, replacement, knee”[MeSH Terms] OR ((“arthroplasty”[All Fields] AND “replacement”[All Fields]) AND “knee”[All Fields])) OR “knee replacement arthroplasty”[All Fields]) OR ((“total”[All Fields] AND “knee”[All Fields]) AND “replacement”[All Fields])) OR “total knee replacement”[All Fields])) OR ((((“arthroplasty, replacement, knee”[MeSH Terms] OR ((“arthroplasty”[All Fields] AND “replacement”[All Fields]) AND “knee”[All Fields])) OR “knee replacement arthroplasty”[All Fields]) OR (“knee”[All Fields] AND “arthroplasty”[All Fields])) OR “knee arthroplasty”[All Fields])) OR ((((“arthroplasty, replacement, knee”[MeSH Terms] OR ((“arthroplasty”[All Fields] AND “replacement”[All Fields]) AND “knee”[All Fields])) OR “knee replacement arthroplasty”[All Fields]) OR (“knee”[All Fields] AND “replacement”[All Fields])) OR “knee replacement”[All Fields])) OR (“TKA”[Title/Abstract] OR “TKR”[Title/Abstract])) AND “valgus”[All Fields] OR “Joint Deformities, Acquired”[Mesh])

**Table 2:** Inclusion and exclusion criteria

Inclusion	
1	Valgus deformities > 10 degrees
2	Intervention: Primary total knee arthroplasty
3	Intervention: peroneal nerve release
4	Articles reporting soft tissue release procedures and pre-and postoperative clinical outcomes (e.g., measurements of alignments and common peroneal nerve palsy)
5	Prospective or retrospective study design.
6	Articles written in English or Dutch.
Exclusion	
1	Valgus deformities < 10 degrees
2	Genu varus, recurvatum, neutral or mixed alignment populations
3	Previous knee surgery, unicompartimental knee arthroplasties or revisions
4	Double publication of the same cohort
5	Systematic reviews, cadaver studies, case reports or studies < 1980

### Data extraction

Data were extracted from each study by the first author (X) and collected in Microsoft Excel 2020 (Microsoft Corp., Redmond, WA, USA). Data of each article that was collected, included first author, study design (pro- or retrospective), study characteristics (year of publication, country, number of knees and patients), patient characteristics (age, gender, body mass index [12], the ratio of osteoarthritis (OA) or rheumatoid arthritis and follow-up), inclusion and exclusion criteria, alignment details (classification type, pre-and postoperative mechanical axis, anatomical axis (aFTA), range of motion and flexion contracture angle), peroneal nerve release, arthroplasty (surgical approach, implant design), lateral soft tissue release (technique, sequence and affected ligaments), osteotomies, common peroneal nerve palsy (duration, preoperative valgus alignment), other complications (residual valgus alignment). In case of unavailable or unspecified information, the authors were contacted and asked to provide the missing information.

### Methodological quality assessment

The quality of the non-randomized studies was assessed by the first author (X), utilizing the Methodological Index for Non-Randomized Studies (MINORS) [44]. In case of any doubt, the second reviewer (X) was consulted to determine the quality of the study. The outcome of the index per study is stated in Table 3.

**Table 3:** Study characteristics

Study and year	Country	Study design	Patients (knees)	Gender, female (%)	Age in years^a^ (mean ± SD)	Patients with OA (%)	Preop. aFTA^a^ (mean ± SD)	Postop. aFTA^a^ (mean ± SD)		preop. FC^a^ (mean ± SD)	CPNP, cases (%)	Soft tissue release in sequence of structures (% of knees)	Follow-up years (mean ± SD^a^)	MINOR score
**No peroneal nerve release – with soft tissue release**														
Greenberg et al. 2020	USA	Retr	95 (104)	–	73 ± 8	n/a	16.2 ± 5.6	5.4 ± 2.8		3.0 ± 6.5	3 (2.9%)	1) ITB subperiosteal (100%) 2) PLC transverse (n/a) 3) POP transverse (n/a)	1.8 ± 1.3	17/24
Li et al. 2020	China	Pros	30 (35)	28 (93)	64.8 ± 8	30 (100%)	20.39 ± 7.79	7.0 ± 2.3		6.4 ± 9.0	1 (2.9%)	1) ITB pie-crusting (100%) 2) LCL transverse (100%) 3) POP transverse (n/a) 4) PLC (n/a)	3.9 ± 2.4	10/16
Ren et al. 2020	China	Retr	58 (61)	47 (81)	65 ± 8	49 (84%)	30.6 ± 6.3	7.3 ± 2.2		0 ± 0	4 (6.6%)	1) ITB (100%) 2) LCL transverse (100%) 3) POP transverse (3.2%)	10.5 ± 2.4	10/16
Cheng et al. 2020	China	Retr	56 (56)	31 (55)	62 ± 3.7	22 (39%)	21.4 ± 5.4	2.6 ± n/a		n/a	0 (0%)	1) ITB subperiosteal (100%) 2) LCL pie-crusting (8.9%) 3) PCL pie-crusting (8.9%)	1 ± 0	10/16
Matar et al. 2019	UK	Retr	104 (110)	87 (84)	68.7 ± 9.2	85 (82%)	18.6 ± 7.5	3.8 ± 2.0		n/a	0 (0%)	1) ITB subperiosteal (100%) 2) POP (28.3%)	5.5 ± 2.4	11/16
Guo et al. 2018	China	Retr	31 (31)	29 (94)	66.5 ± n/a	31 (100%)	21.7 ± 4.6	7.7 ± 1.7		n/a	0 (0%)	Release of the ITB (100%), PLC (100%), LCL (100%)	0.8 ± 0.1	16/24
Jaju et al. 2018	India	Pros	32 (32)	22 (69)	62.7 ± 6.9	9 (28%)	18.59 ± 8.32	3.7 ± 0.9		8.25 ± 4.38	0 (0%)	1) PLC transverse (100%) 2) ITB pie-crusting (47%)	1.9 ± 0.7	11/16
Boettner et al. 2016	USA	Retr	164 (181)	130 (79)	67 ± 9.3	164 (100%)	14.2 ± 4.9	5.2 ± 2.8		4.8 ± 7.3	1 (0.6%)	1) ITB transverse (100%), PLC transverse (100%) and LCL transverse (100%)	2.0 ± 1.0	12/16
Zhou et al. 2014	China	Retr	32 (37)	20 (63)	57.2 ± 6.1	22 (69%)	32.72 ± 9.68	4.89 ± 0.9		−0.78 ± 2.49	3 (8.1%)	1) PLC pie-crusting (100%) 2) LCL pie-crusting (100%) 3) POP transverse (24%)	10 ± 0.7	11/16
Satish et al. 2013	India	Pros	27 (32)	17 (63)	54 ± 5.4	10 (37%)	25.4 ± 12	4 ± 2.5		n/a	2 (6.3%)	1) ITB subperiosteal (100%) 2) PLC transverse (59.3%) 3) POP (21.8%) + LCL (21.8%)	5.0 ± 1.2	12/16
Chechik et al. 2012	Israel	Retr	42 (51)	38 (91)	72.7 ± 8.3	31 (74%)	17.5 ± 4.6	6.3 ± 2.2		[−1] ± 3	1 (2.0%)	1) Transverse release of the PLC (100%)	3.5 ± 1.9	10/16
koskinen et al. 2011	Finland	Retr	48 (52)	46 (96)	66 ± 11.1	37 (77%)	23 ± 8.0	7 ± 5.6		9 ± 5.6 (in 25 knees)	1 (1.9%)	1) ITB pie-crusting (61.5%) 2) POP (40.4%) 3) LCL (18.6%) 4) PLC (5.8%)	9.0 ± 3.6	11/16
Rajgopal et al. 2011	India	Retr	53 (78)	34 (64)	74 ± 10	16 (30%)	20.0 ± 7.2	5.0 ± 0.8		N/A	2 (2.6%)	1) ITB pie-crusting (100%) 2) POP pie-crusting (2.6%)	10.0 ± 1.2	9/16
Apostolo-poulos et al. 2010	Greece	Pros	24 (24)	7 (29)	72 ± 5.6	17 (71%)	23 ± 4.9	5.5 ± 1.3		11 ± 4.3 (in 9 knees)	0 (0.0%)	1) ITB subperiosteal (100%) 2) LCL and POP subperiosteal (100%) 3) PLC (37.5%)	11.5 ± 1.8	11/16
Boyer P et al. 2008	France	Retr	63 (63)	56 (89)	56.9 ± 11.9	43 (68%)	14.7 ± 3.7	1.0 ± 0.4		n/a	1 (1.6%)	1) ITB subperiosteal (100%) 2) LCL and POP subperiosteal (7.9%) 3) PLC (6.3%)	7.0 ± 1.5	10/16
Elkus et al. 2004	USA	Pros	35 (42)	27 (77)	67 ± 12.7	28 (80%)	15.0 ± 5.1	5.0 ± 2.3		7.0 ± n/a	0 (0.0%)	1) PLC + LCL and POP transverse (100%) 2) ITB pie-crusting (100%)	9.0 ± 2.1	13/16
Stern et al. 1991	USA	Retr	98 (134)	83 (85)	68 ± 8.8	88 (90%)	19.0 ± 8.4	7.0 ± 3.4		n/a	5 (3.7%)	1) PLC with LCL and POP transverse (100%) 2) ITB (n/a)	4.5 ± 1.5	11/16
**Peroneal nerve release – with soft tissue release**														
Xu J et al. 2020	China	Pros	30 (34)	21 (70)	70.2 ± 9.3	10 (33%)	31.3 ± 8.0	4.9 ± 2.0		n/a	0 (0%)	1) ITB pie-crusting (100%) 2) PLC pie-crusting (100%) 3) LCL and POP (n/a)	2.25 ± 0.4	12/16
Cree et al. 1999	Australia	Retr	7 (7)	–	72 ± n/a	6 (86%)	24.0 ± 7.4	6.0 ± 2.2		n/a	1 (14.2%)	Not noted	0.6 ± 0.4	9/16
Study	Country	Study design	Patients (knees)	Gender, female (%)	Age in years^a^ (mean ± SD)	Patients with OA (%)	Preop. aFTA^a^ (mean ± SD)	Postop. aFTA^a^ (mean ± SD)	preop FC^a^ (mean ± SD)	CPNP cases (%)	Soft tissue release in sequence of structures (%)	Osteo-tomy (% of knees)	Follow-up years (mean ± SD^a^)	MINOR score
**No peroneal nerve release – with osteotomies and soft tissue release**														
Raut et al. 2020	UK	Retr	23 (25)	15 (65)	68 ± 11.4	14 (61%)	20 ± 4.3	4 ± 1.3	n/a	0 (0%)	1) ITB subperiosteal (100%) 2) POP (100%)	LFEO (100%)	5 ± 3.8	10/16
Mou et al. 2019	China	RCT	25 (27)	–	63 ± 11	n/a	31.6 ± 8	7.0 ± 2.4	n/a	0 (0%)	1) ITB pie-crusting (100%) 2) PLC (n/a)	MFEO (100%)	4.6 ± 0.9	–
Cheng et al. 2019	China	Pros	15 (16)	14 (93)	67.4 ± 6.2	n/a	n/a	n/a	n/a	0 (0%)	1) ITB pie-crusting (100%) 2) PLC transverse (100%)	MFEO (100%)	2.2 ± 0.7	12/16
Scior et al. 2018	Germany	Pros	98 (98)	71 (72)	71.6 ± 10.5	n/a	14.9 ± 2.7	6.4 ± 0.6	4 ± 3	0 (0%)	1) ITB subperiosteal (100%) 2) PLC transverse (100%)	LFEO (100%)	4.5 ± 1	9/16
Conjeski et al. 2018	USA	Retr	10 (12)	7 (70)	68 ± 13.3	9 (90%)	16.4 ± 4.2	5.5 ± 0.9	n/a	1 (8.3%)	No initial soft tissue release	LFEO (100%)	2.9 ± 2.7	10/16
Strauch et al. 2013	Germany	Pros	27 (27)	–	69.5 ± 13.2	n/a	17.7 ± 5.0	7.2 ± 3.8	n/a	0 (0%)	ITB transverse (100%)	LFEO (100%)	1 ± n/a	9/16
Hadjicostas et al. 2008	Germany	Pros	15 (15)	–	73 ± 4.6	13 (87%)	n/a	n/a	n/a	0 (0%)	Partial release of the PLC (n/a)	LFEO (100%)	2.3 ± 0.9	12/16
Brilhault et al. 2002	France	Pros	13 (13)	12 (92)	73 ± 3.6	12 (92%)	n/a	n/a	n/a	0 (0%)	ITB subperiosteal (100%)	LFEO (100%)	4.7 ± 1.7	11/16

*SD* Standard deviation, *OA* Osteoarthritis, *Preop* Preoperative, *Postop* Postoperative, *FC* Flexion contracture, *aFTA* anatomical tibiofemoral angle, *CPNP* Common peroneal nerve palsy, *MINOR* Methodological Index for Non-Randomized Studies, *LFEO* Lateral femoral epicondyle osteotomy, *MFEO* Medial femoral epicondyle osteotomy

^a^Mean ± SD is transformed if needed according to the statistical analysis section

### Statistical analysis

Means and standard deviations were presented and calculated. Reported medians and ranges were transformed into weighted means and estimated standard deviation by the methods of Hozo et al. [45] and Walter et al. [46].

Heterogeneity was assessed using I^2^ and χ ^2^-tests, where an I^2^ of < 25% is considered low; 25–50% as moderate; > 50% as strong and > 75% as substantially heterogeneous by the methods Higgins et al. [24] In case of substantial heterogeneity between studies (I^2^ > 75%), a qualitative/narrative data extraction was performed [24]. As the heterogeneity of the CPNP incidence was not substantial among studies, data were pooled using a fixed-effect model and weighted on sample size. Because of substantial heterogeneity between perioperative continuous outcomes (alignments), these outcomes were qualitatively/narratively described. Pooled CPNP rates were log-transformed to calculate 95% Confidence Intervals. Chi2 tests were performed to assess differences between sub-groups. A *p*-value < 0.05 was considered statistically significant. The Analysis was conducted using R version 4.0.2 (R Foundation statistical computing, Vienna, Austria) with “Metafor package” (Maastricht University, Maastricht, Netherlands).

## Results

Twenty-seven studies were included, representing 1397 valgus knees. Nineteen studies performed only a lateral STR and 8 an OT, including 2 studies with an MFEO and 6 studies LFEO (Table 3). All OT studies used also a lateral STR except one [31]. Two studies performed a PNR in addition to their STR [8, 40]. There was female predominance (mean 70.4%, range 29–94%). The pooled mean age was 67.2 ± 9.1 (range 54–74) and the mean BMI ranged from 23 to 30 kg/m^2^, but was only reported in 6 studies [8, 17, 30, 33, 36, 47]. Preoperative diagnoses included 746 (59.4%) patients with OA, 219 (17.5%) with rheumatoid arthritis, 27 (2.2%) with posttraumatic OA and 263 (21.0%) had an unknown aetiology. Surgery was performed by a medial parapatellar arthrotomy in 16 and a lateral patellar arthrotomy in 11 studies. The weighted mean follow-up was 4.5 years (range, 0.6–10.5).

### Quality of the studies

One randomized controlled trial [33] and 26 non-randomized studies were included. The non-randomized studies consisted of 11 prospective and 15 retrospective cohorts. To estimate the risk of bias of the non-randomized studies, the MINORS criteria were calculated. Two comparative studies had a mean MINOR score of 16.5 (range 16–17) out of 24. The other 24 studies were non-comparative studies with a mean of 10.7 (range, 9–13) out of 16. Only 3 studies (11.5%) reported a prospective calculation of the sample size.

### Common peroneal nerve palsy ratio

In 27 studies, 26 cases of CPNP were reported over a total of 1397 TKA-V (1.9%) (Table 4). The CPNP ratio was comparable between the studies that performed a PNR in 41 knees and the 17 studies that performed only an STR without PNR in 1123 knees (2.4% vs. 2.1%, *p* = 0.90). The pooled mean age and female predominance between groups was comparable (70 vs. 67 years and 70% vs. 73%). Also, no significant difference in CPNP rate was found between the studies that performed an STR in 1164 knees and an OT in 233 knees (2.2% vs. 0.4%, *p* = 0.11). The pooled mean aga and female predominance between the STR and OT group were (67 vs. 70 years, 71% vs. 78%). The study of Conjeski et al. [31] was responsible for the only CPNP case in the entire OT group (*n* = 233) but was also the single study that used no additional lateral STR and has let the piece of the OT healed in situ without use of internal fixation. Three studies did not explicitly describe CPNP cases in their complication section, therefore it was assumed that CPNP did not occur in those studies [35, 37, 48]. No study clarified if a CPNP case was developed from a patient with posttraumatic OA. Nevertheless, only two studies had patients (11 knees) with posttraumatic OA, where also CPNP cases (7 knees) were reported [15, 16]. Information on whether these CPNP cases occurred in one of these 11 knees was lacking.

**Table 4:** Prevalence of common peroneal nerve palsy

Treatment group	Studies (n)	Patients (No of knees)	CPNP cases (%)	Pooled proportion (%)	95% CI (%)	Heterogeneity I^2^- (%)	*P*-value
Osteotomy	8	226 (233)	1 (0.4%)	0.43	0.01–2.37	0%	
Soft tissue release	19	1029 (1164)	25 (2.2%)	2.15	1.39–3.15	0%	0.11
PNR	2	37 (41)	1 (2.4%)	2.44	0.06–12.86	46%	
No PNR	17	992 (1123)	24 (2.1%)	2.14	1.37–3.16	2%	0.90
Overall	27	1255 (1397)	26 (1.9%)	1.86	1.22–2.72	0%	

*No* Number of, *CPNP* Common peroneal nerve palsy, *PNR* Peroneal nerve release, *CI* Confidence interval

### Soft tissue releases

In the 19 studies that solely performed a lateral STRs, a large variation in released ligaments was present compared with the studies that performed an OT, that mainly released the ITB and PLC. A single study in the OT group performed a POP release [35]. A sub-analysis was performed to approximate the difference in CPNP rate between different specifically released ligaments (Table 5). Between the releases of different ligaments or the manner of those releases (pie-crusting, subperiosteal or transverse), no significant difference in CPNP rate was found. Only studies that reported that all patients underwent a release of a specific ligament were included for sub-analysis. One study was excluded from any analysis due to a lack of data [40].

**Table 5:** Specific ligament release and CPNP ratio

Released ligament	^A^ Studies (n)	Treated knees (%)	CPNP cases, (%)	*P*-value
No ITB releases	4	114 (9.7%)	4 (3.5%)	
ITB release overall	19	1057 (90.3%)	14 (1.3%)	0.09
Pie-crusting	6	232 (21.9%)	3 (1.3%)	
Subperiosteal	9	525 (49.7%)	6 (1.1%)	
Transverse	2	208 (19.7%)	1 (0.5%)	0.66
Unclear ^A^	2	92 (8.7%)	0 (0.0%)	
No PLC releases	7	326 (33.2%)	7 (2.1%)	
PLC release overall	10	656 (66.8%)	10 (1.5%)	0.60
Pie-crusting	2	71 (10.8%)	3 (2.4%)	
Transverse	7	553 (84.3%)	7 (1.3%)	0.07
Unclear ^A^	1	32 (4.9%)	0 (0.0%)	
No LCL releases	18	608 (52.7%)	7 (1.2%)	
LCL release overall	8	545 (47.3%)	14 (2.6%)	0.08
No POP releases	14	700 (75.7%)	9 (1.3%)	
POP release overall	4	225 (24.3%)	5 (2.2%)	0.35

*CPNP* Common peroneal nerve palsy, *ITB* Iliotibial band, *PLC* Posterior lateral capsule, *LCL* Lateral collateral ligament, *POP* Popliteus tendon. A: Studies that released a lateral soft tissue, but without specifying in which manner this was performed

Studies that performed a specific ligament release on only a part of the total study population were excluded for analysis. One study [40] was excluded for analysis due to lack of data

### Pre and postoperative alignments

The overall weighted mean pre- and postoperative aFTAs was 19.5 ± 8.4 and 5.3 ± 2.7 degrees (Table 6). The PNR group was the only group with a considerable larger weighted mean preoperative aFTA (30.1 ± 1.3), and therefore, a larger Valgus Correction Angle (VCA) (25.0°). All weighted postoperative aFTAs were comparable between the different groups. Due to a high heterogeneity between studies (I^2^ > 0.80), no statistical analysis could be performed. Regarding to the CPNP cases, 4 studies reported the individual preoperative aFTA of 4 CPNP cases, which were 19° [49], 25° [47], 26° [31] and 38° [40]. Flexion contracture angles were reported in 11 studies (41%), with an overall weighted mean of 4.1 ± 4.8°. No individual flexion contractures of CPNP cases were reported in the studies.

**Table 6:** Pre and -postoperative alignments

	Studies ^A^	Patients (No of knees)	Mean preoperative aFTA° ± SD	Mean Postoperative aFTA° ± SD	mean VCA^B^
Osteotomy	5	189 (190)	18.5 ± 5.7	6.2 ± 0.7	12.3
Soft tissue releases	19	1029 (1164)	18.6 ± 6.2	5.2 ± 2.5	13.4
PNR	2	37 (41)	30.1 ± 1.3	5.1 ± 0.3	25.0
No PNR	17	992 (1123)	19.3 ± 6.1	5.2 ± 2.5	14.1
Overall	24	1212 (1353)	19.5 ± 8.4	5.3 ± 2.7	14.2

*CPNP* Common peroneal nerve palsy, *VCA* Valgus correction angle, *aFTA* Anatomical femorotibial angle

No statistical analysis is performed due to a substantial heterogeneity between the studies. All studies are weighted by the number of operated knees. Three study were excluded due to lack of data [29, 30, 32]. Valgus correction angle is calculated by postoperative aFTA minus preoperative aFTA

## Discussion

In this systematic review, the most important finding was the overall CPNP ratio of 1.9% after TKA-V. No significant differences in CPNP rate were found between TKA-V with and without PNR (2.4% vs 2.1%), between TKA-V with lateral STR (2.2%) or with OT (0.4%) and between the releases of different ligaments or the manner of those releases (pie-crusting, subperiosteal or transverse).

The obtained overall CPNP ratio of 1.9% in this study falls within the known range of TKA-V (0.3% - 9.5%) [3, 9–17]. Other systematic review reported a range of 0.01% to 4.3%, like the one of Carender et al. [2] and Rodríguez-Merchán et al. [50]. Currently, controversy still exists related to valgus deformity being a predisposing factor of CPNP. Studies that have investigated the location of the CPN, indicate that the CPN can be jeopardized by a direct injury due to pie-crusting or a transverse release of the ITB or PLC in well-aligned and valgus knees [19, 20, 51]. However, in our review, we could not confirm the increase of risk to injure the CPN by different ligament releases. Therefore, our results may support the theory that most CPNPs probably occur due to postoperative mechanical damage, like traction and compression, instead of a direct injury. Besides, one large registry-based study by Christ et al. [3], including 383,060 primary TKA procedures, found that preoperative valgus alignments increase the risk of developing a CPNP significantly (OR 4.19). Also, Idusuyi et al. [4], found a relative risk of CPNP 12 times greater for patients with a 12° or more valgus deformity. Both studies did not find an association between CPNP and flexion contractures. However, according to Christ et al. [3], this may be because the diagnosis code for flexion contractures is not consistently noted as that of valgus deformities in their registry. Therefore, the data may be biased. Other studies, like Park et al. [12] and Schinsky et al. [52] found an overall incidence of 0.53% and 1.3% but did not find any relation between valgus deformities and CPNP. However, all these studies used mixed preoperative alignments. Therefore, it is difficult to compare the CPNP ratio of this systematic review, with the incidence of other reviews or studies. Eventually, knowing that larger previous studies showed an increase in CPNP incidence in valgus knees, we would advise clinicians to perform a TKA-V with extra care. This would enable PNR as an option for severe valgus deformities since the procedure is minimally invasive and may lead to preventing CPNP. However, this current review did not find a significant difference in CPNP incidence between the studies that performed a TKA-V with and without PNR. Regarding the 2 studies utilizing a PNR, the study of Cree et al. [40] is a small retrospective study and the recent study of Xu et al. [8] is a small prospective study, both studies performed the same surgical technique. The studies together account for a population of 41 knees in which 1 developed a CPNP. Focussing on that single CPNP case, the study of Cree et al. [40] mentioned that the CPN remained too tight after an extensive PNR due to a vast preoperative aFTA of 38° and VCA of 30°. Therefore, this CPNP developed assumably due to the postoperative stretch of the CPN. Furthermore, comparing the perioperative alignments of the 2 PNR studies with the non-PNR studies show that the preoperative aFTA of the 2 PNR studies is substantially bigger (30.1°. vs 19.3°). However, it is difficult to compare these alignments without any statistical analysis due to the high heterogeneity between the studies. It is noticeable that the 4 individual CPNP cases that are mentioned in the 4 studies, all have a higher preoperative aFTA (19° [49], 25° [47], 26° [31] and 38° [40]) than the overall mean aFTA of all the studies in this review.

In the end, the results in this review suggest that a PNR procedure is not effective. However, it is difficult to assume such an interpretation because only two small sample sized studies were found that used a PNR prior to TKA and met the inclusion criteria of our systematic review [8, 40]. Future research should further investigate PNR in larger study populations and preferably with a comparison group, which would make it easier to interpret results.

Like all studies, some limitations need to be discussed. Firstly, the considerable heterogeneity between the included studies, possibly caused by our caution to minimize selection bias in including studies for this review. However, due to the low incidence of CPNP and the focus on valgus deformities, a comprehensive literature search was needed. Secondly, the review lacks important detailed information about the individual cases who developed a CPNP in the studies. In addition, preoperative data of the knees, like knee extension angles and stress radiographs to assess whether there is a fixed valgus deformity are missing in most studies. Therefore, it is important for future studies to specify the manner and degree of the surgeries and to comprehensively note the pre and postoperative data of the knees. Thirdly, the scarce of studies investigating a PNR is an insurmountable problem, which made it impossible to draw conclusions. However, this review provides a basis for future work investigating PNR in valgus knees to prevent CPNP.

## Conclusion

To our knowledge, this is the first systematic review that provides insight into the current literature about preventing CPNP with a PNR after TKA-V. An overall CPNP ratio of 1.9% in valgus knees after TKA was found. There was no direct evidence that using a PNR would be more effective than not using a PNR in preventing a CPNP. However, it was impossible to draw conclusions, due to the scarce amount of literature. Therefore, larger studies comparing TKA-V with and without PNR are needed to appropriately define the efficiency of a PNR. This systematic review is the first step in this regard.

## Supplementary Information

**Additional file 1.** **Figure S1**. PRISMA flow diagram. The flow diagram of study selection per guidelines from the Preferred Reporting Items for Systematic Reviews and Meta-Analyses (PRISMA) group.

## Abbreviations

TKA: Total Knee Arthroplasty
TKA-V: Total Knee Arthroplasty in Valgus deformities of > 10° aFTA
STR: Soft Tissue Release
OT: Osteotomy
ITB: Iliotibial Band
LCL: Lateral Collateral Ligament
PLC: Posterolateral Capsule
POP: Popliteus tendon
CPNP: Common Peroneal Nerve Palsy
CPN: Common Peroneal Nerve
PNR: Peroneal Nerve Release
aFTA: anatomic FemoroTibial Angle
VCA: Valgus Correction Angle
LFEO: Lateral Femoral Epicondyle Osteotomy
MFEO: Medial Femoral Epicondyle Osteotomy
