# Supplementary figures and images for: Common peroneal nerve palsy after TKA in valgus deformities; a systematic review
Source: J Exp Orthop. 2022 Jan 20;9:12. doi: 10.1186/s40634-021-00443-x (PMC8776926; doi:10.1186/s40634-021-00443-x)

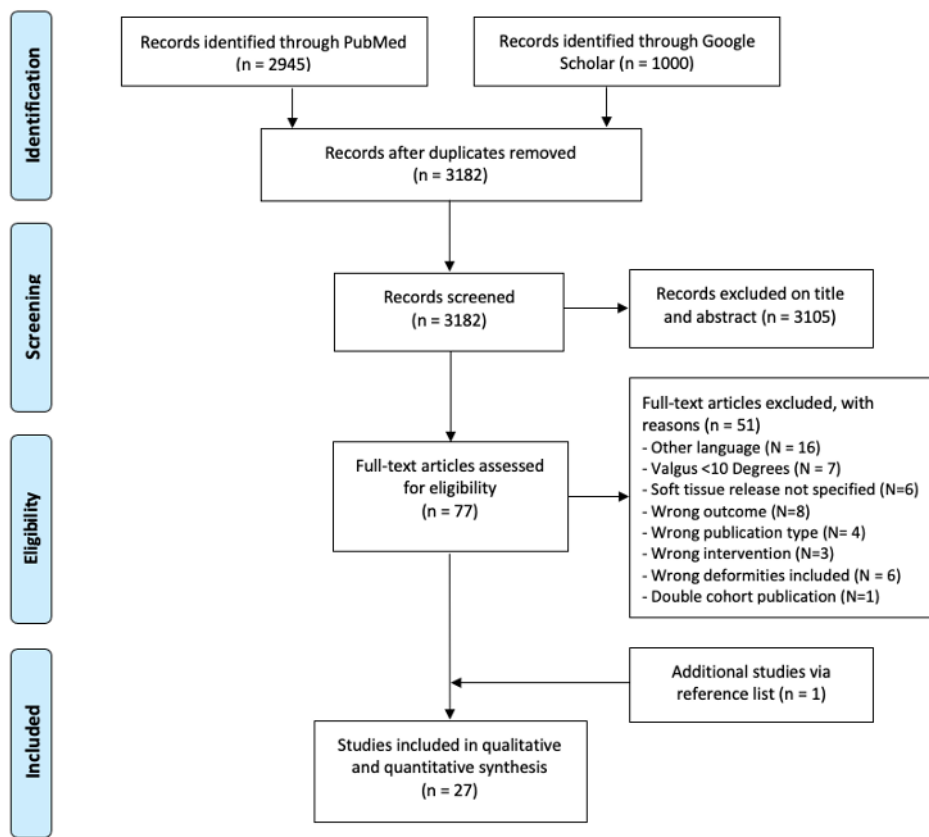

Supplement: Supplementary file 1 — Additional file 1. Figure S1. PRISMA flow diagram. The flow diagram of study selection per guidelines from the Preferred Reporting Items for Systematic Reviews and Meta-Analyses (PRISMA) group. [file 40634_2021_443_MOESM1_ESM.pdf]
